# Supplementary material for: Awareness and knowledge associated to Human papillomavirus infection among university students in Morocco: A cross-sectional study
Source: PLoS One. 2022 Jul 8;17(7):e0271222. doi: 10.1371/journal.pone.0271222 (PMC9269923; doi:10.1371/journal.pone.0271222)
Supplement: S2 Table — (DOCX) [file pone.0271222.s002.docx]

**Table 1. Factors associated with HPV awareness and knowledge on HPV, using univariate and multivariate logistic regression analysis.**

| Variables | HPV Awareness | | | | HPV Knowledge | | | |
| --- | --- | --- | --- | --- | --- | --- | --- | --- |
|  | Univariate analysis | | Multivariate analysis | | Univariate analysis | | Multivariate analysis | |
|  | OR (CI 95%) | *p-value* | aOR (CI 95%) | *p-value* | OR (CI 95%) | *p-value* | aOR (CI 95%) | *p-value* |
| Age categories |  |  |  |  |  |  |  |  |
| 17-21 | 1 |  | 1 | 0.096 | 1 | 0.865 | - | - |
| 22- 28 | 2.62 (1.32-5.17) |  | 1.85 (0.89- 3.83) |  | 0.89 (0.23-3.36) |  |  |  |
| Gender |  |  |  |  |  |  |  |  |
| Men |  |  | - | - | 1 | 0.019γ | 1 | 0.047ϯ |
| Women | 1.13 (0.62- 2.05) |  |  |  | 4.58 (1.29-16.26) |  | 3.76 (1.01-13.92) |  |
| Marital status |  |  | - | - |  |  | - | - |
| Single |  |  | - | - | 1 | 1.000 | - | - |
| Married | 2.27 (0.24-20.74) |  |  |  | 0.00 (0.00^NS^) |  |  |  |
| Employed |  |  |  |  |  |  |  |  |
| Yes | 1 |  | - | - | 1 | 0.347 | - | - |
| No | 0.32 (0.03- 3.23) |  |  |  | 0.00 (0.00^NS^) |  |  |  |
| Geographic origin |  |  |  |  |  |  |  |  |
| Urban | 1 | 0.575 | - | - | 1 |  | - | - |
| Rural | 0.81 (0.40- 1.65) |  |  |  | 0.49 (0.11-2.15) | 0.347 |  |  |
| Level of education |  |  |  |  |  |  |  |  |
| Undergraduate |  | 0.000γ | 1 | 0.000ϯ | 1 | 0.048γ | 1 | 0.145 |
| Graduate | 6.83 (3.42- 13.66) |  | 4.04 (1.92- 8.52) |  | 3.49 (1.01-12.05**)** |  | 2.65 (0.71- 9.81) |  |
| Curriculum |  |  |  |  |  |  |  |  |
| Non-biology |  | 0.000γ | 1 | 0.000ϯ | 1 | 0.999 | - | - |
| Biology | 6.77 (2.82- 16.27) |  | 5.20 (2.12-12.73) |  | 0.00 (0.00^NS^) |  |  |  |

% percentage. HPV=Human Papillomavirus, CI= Confidence interval. OR= Odds ratio. aOR= Adjusted Odds ratio. Ref= Reference category. NS= Not significant. γ =P<0.20 (significant in the univariate model). ϯ =P<0.05 (significant in the multivariate model).
