# Supplementary material for: Awareness and knowledge associated to Human papillomavirus infection among university students in Morocco: A cross-sectional study
Source: PLoS One. 2022 Jul 8;17(7):e0271222. doi: 10.1371/journal.pone.0271222 (PMC9269923; doi:10.1371/journal.pone.0271222)
Supplement: Attachment — Submitted filename: Manuscript.docx [file pone.0271222.s003.docx]

**Full title: Knowledge and Awareness toward Human Papillomavirus and Cervical Cancer: A Cross-sectional Study Among University Students in Morocco**

**Short title: Human Papillomavirus and Cervical Cancer**

Nezha EL MANSOURI^1*^; Laila FERRERA^1^; Ahmed KHARBACH^2^; Abderrahmane ACHBANI^1^; Farid KASSIDI^1^; Hanane ROGUA^1^; Sofiane AIT WAHMANE^1^; Ahmed BELMOUDEN^1^; Said CHOUHAM^1^ and Mohamed NEJMEDDINE^1*^.

^1^ Laboratory of Cell Biology and Molecular Genetics (LBCGM), Department of Biology, Faculty of Sciences, Ibn Zohr University of Agadir, MA.

^2^ Laboratory of Biostatistics, Clinical Research and Epidemiology (LBRCE), Faculty of Medicine and Pharmacy of Rabat, Mohammed V University of Rabat, MA

*Corresponding authors:

E-mail: [nezha.elmansouri@edu.uiz.ac.ma](mailto:nezha.elmansouri@edu.uiz.ac.ma) (NEM)

E-mail: [m.nejmeddine@uiz.ac.ma](mailto:m.nejmeddine@uiz.ac.ma)	(MN)

Acknowledgements

We would like to thank all the students that kindly agreed to participate in this study.

**Abstract**

Worldwide, cervical cancer is a real health issue, however, gaps exist in the public’s awareness of the causal role of Human papillomavirus (HPV) in the development of this disease. This study aims to assess the level and the associated factors of awareness and knowledge on HPV among university students in Morocco. A cross-sectional study was conducted with a descriptive and analytical aim, among students attending Ibn Zohr University, in Agadir, Morocco. An interview questionnaire was used to collect information about the participants: demographic data, awareness and level of knowledge on HPV infection, and awareness of cervical cancer. Logistic regression analyses were to determine the associated factors with awareness and level of knowledge on HPV. A total of 479 students participated in this study (mean age 21.82 ± 2.091). Most participants 81.6% (n=391) were aware of cervical cancer, while only 1.5% (n=7) identified HPV as a sexually transmitted infection. Among students, 10.45% (n=48) were aware of HPV but only half of them 47.9% (n=23) confirmed that HPV is associated with cervical cancer, and 60.4% (n=29) showed low knowledge on HPV. Multivariateanalysis revealed that HPV awareness has a strong association with a higher level of study (OR 4.04; 95% CI: 1.92-8.52), and with being a biology student (OR 5.20; 95% CI: 2.12-12.73), while high HPV knowledge was only associated with the female gender (OR 3.76; 95% CI: 1.01-13.92). The data suggest that university students in Morocco did not show sufficient knowledge of HPV infection and its consequences. This supports that earlier incorporation of sexual health education programs, especially related to HPV and cervical cancer, must be implemented in the university to reduce the burden of HPV-associated diseases among the population at risk.

**Keywords:** Human papillomavirus, cervical cancer, awareness, knowledge, University student, Morocco.

# Introduction

The papillomavirus belongs to the family of papillomaviridae [1]. They co-evolved over millions of years with a diverse range of animal hosts as well as humans [2]. Human papillomavirus (HPV) is one of the most common causes of sexually transmitted infections (STIs) among young and sexually active men and women worldwide [3]. HPVs constitute a group of more than 200 different types associated with benign and malignant neoplasms of the skin and mucosal membranes [4]. Among them, forty different HPV types, known to infect the genital system. These HPV types are subdivided into risk low-risk types, which cause mainly genital warts; and risk high-risk types, which were reported to be responsible for almost all cases of cervical cancer (>99.7%) [5,6]. HPV 16 and HPV 18 are the most carcinogenic HPV types, causing approximately 70% of all cervical cancer cases [7]. Three vaccines with a good safety profile were developed and were shown to be an effective primary prevention strategy against the most common HPV strains. Bivalent (HPV‑16 and HPV‑18), and quadrivalent (HPV‑16, HPV‑18, HPV‑6, and HPV‑11), and non-avalent vaccine targets the same HPV types as the quadrivalent vaccine as well as types (31, 33, 45, 52, and 58) [8,9]. HPVs were also shown to be associated with 30–50% of head-and-neck cancers, and cause different types of anogenital cancers [10]. Most HPV infections of the cervix are asymptomatic and more than 90% of detected infections are cleared within two years [11]. However, cervical cancer is mainly a consequence of the persistence of carcinogenic infections of HPV, which are transmitted sexually in early adulthood [12].

Cervical cancer is the fourth most frequently diagnosed cancer in women worldwide, with an estimated 604,000 new cases in 2020, representing 6.5% of all female cancers. It is the fourth leading cause of cancer death in women, with 342,000 deaths worldwide, according to the latest report from the International Agency for Research on Cancer (IARC) in 2020 [13]. This cancer occurs mostly in low and middle-income countries. The highest incidence rates have been recorded in Eastern Africa, while incidence rates are 7 to 10 times lower in North America, Australia/New Zealand, and Western Asia (40.1 per 100,000 in Eastern Africa vs 5.6 per 100,00 in Australia/New Zealand) in 2020 [13].

Cervical cancer is a major health issue in North Africa, in particular in Morocco. Indeed, 2165 new cases were diagnosed in 2020, representing 7.2% of all the female cancers in the country. It also comes in the second position, after breast cancer is the leading cause of death by cancer in women, with 1199 death in 2020 [13].

The high frequency of cervical cancer in Morocco might be due either to the limited access to health care coverage especially in remote areas in the southern regions of the country or to poor awareness and knowledge about this illness as barriers to use preventive methods [14,15]. Despite these statistics, cervical cancer is one of the few preventable, and curable human cancer if detected earlier. It could be prevented by primary prevention (large vaccination programs), and secondary prevention approaches (early diagnosis by screening, and by implementing effective treatment programs). In addition, promising prevention strategies based on educational programs to improve knowledge of the pathogenesis of HPV and cervical cancer [16].

In Morocco, as well as worldwide, very little is known about the awareness of the general population regarding the association between cervical cancer and HPV infection [17,18]. In this present study, we aim to investigate the level and the associated factors of awareness and knowledge on HPV among higher education students, attending Ibn Zohr University in Morocco, at the age of sexual activity.

# Materials and methods

## The study setting and population sample

This cross-sectional study was conducted among students attending Ibn Zohr University (IZU), in Agadir, Morocco. The participants were selected using a simple random selection process. However, the population of students targeted was mainly those attending courses in the Faculty of Sciences. The objective of the study was fully explained to the participants before the data collection and when consent was given, subjects were included in the study. A detailed interview questionnaire was administered to all participants to collect the information needed for our research. In case of doubt, our team members helped clarify questions for the students.

## The questionnaire

The questionnaire was divided into three main sections. The first section: was designed to collect the socio-demographic information of the participants (i.e., age, gender, marital status, employment status, geographic origin, level of study, and curriculum).

In the second section: participants were first asked to answer the following open question (Q1): Do you know any sexually transmitted infections (STIs)? This was followed by an open-ended question with “Yes” or “No” response options (Q2): Have you ever heard of cervical cancer? To assess the students’ awareness of HPV infection, we asked them to answer another open-ended question with “Yes” or “No” response options (Q3): Have you ever heard of the human papillomavirus (HPV)?

Only the participants that responded “Yes” to the question above (Q3) were considered aware of HPV and were asked to complete section 3 related to knowledge on HPV infection. This section included five open-ended questions with Yes/No/Don’t know response options as follows: Is HPV infection symptomatic? (Q4); is HPV infection associated with any disease? (Q5); Is there any association between HPV and cervical cancer? (Q6); Do you think that HPV infection is the main cause of cervical cancer? (Q7); Do you know any prevention strategies against HPV infection? (Q8). We allocated one point for each correct answer, and zero for the incorrect or the “Don’t know” response. The total score was calculated by adding up all scores for the five questions (Q4 to Q8). The scores higher or equal to three was considered, “High knowledge”; while a score lower than three was considered, “Low knowledge”. The data were archived as digital files.

## The statistical analysis

Descriptive statistics include frequencies and percentages for categorical variables. Mean and standard deviation (SD) for continuous variables were calculated. Chi-square (χ^2^) and Fisher’s exact analyses were used to identify the associated sociodemographic variables to HPV awareness, knowledge on HPV, and cervical cancer awareness. P-values <0.05 were considered significant.

Odds ratio (OR), *p-value* and 95% confidence intervals (CI) were obtained using logistic regression models. Univariate and multivariate logistic regression analyses were used to determine the significant associations between the socio-demographic variables and awareness of HPV infection, and the level of knowledge on HPV infection. Variables that exhibited statistical significance at the 0.2 level in the univariate analysis were included in the multivariate analysis using a logistic regression model adjusted by potential confounders [19]. Statistical significance was defined as *p-value* < 0.05.

The collected data were analyzed using statistical analysis software (IBM SPSS statistics version 13.0, New York, NY).

## Ethics approval

The study has been approved by the Bioethics consultative commission of the Faculty of Sciences of Agadir (BECC-FSA Ref. No: FCR-CS-05/2021-0001).

This research was fully approved by the Bioethics consultative commission of the Faculty of Sciences of Agadir (BECC-FSA Ref. No: FCR-CS-05/2021-0001). Informed consent was obtained from each participant included in this study.

# Results

## The characteristics of the population

A total of 479 students were surveyed in this study, with a mean age of 21.82 ± 2.091, ranging from 17 to 28 years. Among the participants, 50.70% (n= 243) were males, and 49.3% (n=236) were females. The majority of the interviewees were full-time students 99.20% (n=475), single 99.00% (n=474), and came from urban areas 73.7% (n=353). More than half of the participants 261 (54.50%) were pursuing their studies in biology curricula, and the undergraduate students accounted for 89.80% (n=430) of all the participants (Table1).

## Awareness toward HPV infection

The results showed that the acquired immune deficiency syndrome (AIDS) is the most cited sexually transmitted infection (STI) among all the STIs mentioned by the students (Q1). Among the respondents 95.80% (n=459) mentioned AIDS in the first place, followed by syphilis 17.30% (n=83), and hepatitis B which was mentioned by only 6.70% (n=32) of the students. Other STIs have been mentioned such as herpes 0.80% (n=4), *Chlamydia* *spp.* 0.60% (n=3) and *Trichomonas spp.* (0.20% (n=1). Regarding Human papillomavirus (HPV) infection, we found that only 7 students (1.50%) knew that HPV infection is an STI.

Students were also asked if they knew about cervical cancer (Q2). Surprisingly, the vast majority of them have already heard about cervical cancer (81.6% (391 out of 479)), and significant differences were reported between the group who had heard of cervical cancer and the group who hadn’t heard of it in relation to: gender (*p*= 0.000) and curriculum (*p*= 0.000) [Table 1]. However, only 10.45% (n= 48) replied to have already heard about HPV (Q3). These students were conscious of HPV infection.

Differences in HPV awareness groups by age (*p*= 0.004), level of study (*p*= 0.000) and curriculum (*p*= 0.000) were significant (Table 1).

To investigate the association between the awareness about HPV infection and some key features of the population, we performed univariate logistic regression statistical analyses (Table 2).

This analysis showed that HPV awareness correlated positively with the age of the participants, their curriculum, and their level of study.

**Table 1: Sociodemographic characteristics of the study sample, and by HPV awareness, knowledge on HPV, and cervical cancer awareness.**

| Variables | Number (%)  n=479 | HPV Awareness n=479  Have you ever heard of HPV? | | | Knowledge on HPV n=48 | | | Cervical Cancer Awareness n=479  Have you ever heard of CC? | | |
| --- | --- | --- | --- | --- | --- | --- | --- | --- | --- | --- |
|  |  | Yes  n (%) | No  n(%) | *p-value* | Low n(%) | High  n(%) | *p-value* | Yes  n(%) | No  n(%) | *p-value* |
| Age (mean ± SD)  17-21  22- 28  Gender  Men  Women  Marital status  Single  Married  Employed  Yes  No  Geographic origin  Urban  Rural  Level of study  Undergraduate  Graduate  Curriculum  Non-biology  Biology | 21.82 ± 2.091  213 (44.50)  266 (55.50)  243 (50.70)  236 (49.30)  474 (99.00)  5 (1.00)  4 (0.8)  475 (99.20)  353 (73.70)  126 (26.30)  430 (89.80)  49 (10.20)  261 (54.50)  218 (45.50) | 12 (5.6)  36 (13.5)  23 (9.5)  25 (10.6)  47 (9.9)  1 (20.0)  1 (25.0)  47 (9.9)  37 (10.5)  11 (8.7)  31 (7.2)  17 (34.7)  6 (2.8)  42 (16.1) | 201 (94.4)  230 (86.5)  220 (90.5)  211 (89.4)  427 (90.1)  4 (80.0)  3 (75.0)  428 (90.1)  316 (89.5)  115 (91.3)  399 (92.8)  32 (65.3)  212 (97.2)  219 (83.9) | 0.004*  0.681*  0.412*  0.345*  0.574*  0.000*  0.000* | 7 (58.3)  22 (61.1)  18 (78.3)  11 (44.0)  29 (61.7)  0 (0.0)  0 (0.0)  29 (61.7)  21 (56.8)  8 (72.7)  22 (71.0)  7 (41.2)  23 (54.8)  6 (100.0) | 5 (41.7)  14 (38.9)  5 (21.7)  14 (56.0)  18 (38.3)  1 (100.0)  1 (100.0)  18 (38.3)  16 (43.2)  3 (27.3)  9 (29.0)  10 (58.8)  19 (45.2)  0 (0.0) | 1.000*  0.015*  0.396*  0.396*  0.488*  0.044*  0.068* | 167 (78.4)  224 (84.2)  181 (74.5)  210 (89.0)  386 (81.4)  5 (100.0)  4 (100.0)  387 (81.5)  290 (82.2)  101 (80.2)  346 (80.5)  45 (91.8)  163 (74.8)  228 (87.4) | 46 (21.6)  42 (15.8)  62 (25.5)  26 (11.0)  88 (18.6)  0 (0.0)  0 (0.0)  88 (18.5)  63 (17.8)  25 (19.8)  84 (19.5)  5 (8.2)  55 (25.2)  33 (12.6) | 0.103*  0.000*  0.590*  1.000*  0.620*  0.051*  0.000* |

n= number of participants. % percentage. HPV= Human Papillomavirus. CC= Cervical Cancer. *= The Chi-square test or Fisher’s exact test.

The awareness about HPV infection was higher in the population of the students in the age ranged between 22 and 28 years, compared to the aged between 17 and 21 years old (13.5% *vs* 5.6%) (OR= 2.62; 95% CI= [1.32-5.17]; *p*= 0.005). In addition, graduate students were more likely to be aware of HPV infection compared to undergraduate students (OR 6.83; 95% CI= [3.42-13.66] *p*=0.000). Students with a biology curriculum knew about HPV compared to non-biology curriculum students (16.1% *vs* 2.8%) (OR= 6.77; 95% CI= [2.82-16.27]; *p*= 0.000). To confirm these results, we entered the most significant variables into a multivariate model. This model showed that the high level of study (OR=4.04; 95% CI= [1.92-8.52]; *p*=0.001), and the nature of the curriculum (OR=4.92; 95% CI= [2.12-12.73]; *p*= 0.001) remained significantly associated factors with HPV awareness (Table 2).

## Knowledge about HPV infection

The results showed that among the students who previously have heard about HPV infection, 60.4% (n=29) knew that HPV infection is asymptomatic (Q4). Only 22 out of 48 students (45.8%) knew that HPV infection can be associated with other diseases (Q5). The association between HPV and cervical cancer was confirmed by 47.9 % (n=23) of the participants (Q6). More than half 54.2% (n=26) responded that infection with HPV is the leading cause for the development of cervical cancer (Q7). Finally, the majority of the students (68.8% (n=33)) were unable to suggest any prevention strategy against the HPV infection (Q8). Regarding HPV knowledge, the participants could be divided into two groups: Low knowledge (60.4% (n=29)) and high knowledge (49.6% (n= 19)), and significant differences were found between the group with low HPV knowledge and the group with high HPV knowledge based on: Gender (*p*= 0,015) and level of study (*p=* 0.044) (Table 1).

The univariate logistic regression analysis revealed that knowledge about HPV was associated significantly with gender and level of study. Females were more likely to have higher knowledge about HPV compared to males (56.0% *vs* 21.7%) (OR= 4.58; 95% CI= [1.29-16.26]; *p*= 0.019) (Table 2). Education level had a positive effect on HPV knowledge, graduate students were more likely to have better knowledge of HPV compared to undergraduate ones (OR=3.49; 95% CI= [1.01-12.05]; *p*= 0.048). The multivariate analysis showed that only gender remained significantly associated factor with HPV knowledge (OR= 3.76; 95% CI= [1.01-13.92]; *p*= 0.047) (Table 2)

**Table 2:** **Factors associated with HPV awareness and knowledge on HPV, using univariate and multivariate logistic regression analysis.**

| Variables | HPV Awareness | | | | HPV Knowledge | | | |
| --- | --- | --- | --- | --- | --- | --- | --- | --- |
|  | Univariate analysis | | Multivariate analysis | | Univariate analysis | | Multivariate analysis | |
|  | OR (CI 95%) | *p-value* | aOR (CI 95%) | *p-value* | OR (CI 95%) | *p-value* | aOR (CI 95%) | *p-value* |
| Age (mean ± SD)  17-21  22- 28  Gender  Men  Women  Marital status  Single  Married  Employed  Yes  No  Geographic origin  Urban  Rural  Level of study  Undergraduate  Graduate  Curriculum  Non-biology  Biology | Ref  2.62 (1.32-5.17) | 0.005γ | Ref  1.85 (0.89- 3.83) | 0.096 | Ref  0.89 (0.23-3.36) | 0.865 | - | - |
|  | Ref  1.13 (0.62- 2.05) | 0.681 | - | - | Ref  4.58 (1.29-16.26) | 0.019γ | Ref  3.76 (1.01-13.92) | 0.047ϯ |
|  | Ref  2.27 (0.24-20.74) | 0.467 | - | - | Ref  0.00 (0.00^NS^) | 1.000 | - | - |
|  | Ref  0.32 (0.03- 3.23) | 0.340 | - | - | Ref  0.00 (0.00^NS^) | 1.000 | - | - |
|  | Ref  0.81 (0.40- 1.65) | 0.575 | - | - | Ref  0.49 (0.11-2.15) | 0.347 | - | - |
|  | Ref  6.83 (3.42- 13.66) | 0.000γ | Ref  4.04 (1.92- 8.52) | 0.000ϯ | Ref  3.49 (1.01-12.05**)** | 0.048γ | Ref  2.65 (0.71- 9.81) | 0.145 |
|  | Ref  6.77 (2.82- 16.27) | 0.000γ | Ref  5.20 (2.12-12.73 | 0.000ϯ | Ref  0.00 (0.00^NS^) | 0.999 | - | - |

% percentage. HPV=Human Papillomavirus, CI= Confidence interval. OR= Odds ratio. aOR= Adjusted Odds ratio. Ref= Reference category. NS= Not significant. γ =P<0.20 (significant in the univariate model). ϯ =P<0.05 (significant in the multivariate model).

# Discussion

Very limited reports investigated the awareness of STIs, particularly the HPV infection and its association with cervical cancer among higher education students in Morocco, despite this part of the population was the most concerned by such issues.

Cervical cancer is a real health issue in Morocco, mainly because of the low coverage of screening programmes, and the difficulties in implementing free HPV vaccination in the country. In addition, there is a big lack of knowledge among the general public, on what causes cervical cancer [20,21]. Awareness alone is not sufficient to tackle this major health problem. However, it can make a huge difference in preventing and controlling cervical cancer’s rising death and cases.

As a first approach to assess the awareness of HPV infection, the students were asked to indicate which STIs they already knew. This question was mainly asked to assess their prerequisite on sexual health and to find out if the students can identify HPV infection among STIs. We showed that the majority of students have little knowledge concerning STIs. In addition, very few of them knew that HPV infection is STI (1.50%). However, a high proportion of them (95.8%) were aware that AIDS is STI. AIDS was the best known STI, which might be the result of the massive educative programmes through the media, schools, and health professionals which enhanced awareness of the general population about it. Similarly, it was reported in Egypt that 95.9% of the students knew about AIDS, but they showed a poor knowledge about sexual health issues [22]. By contrast, students in the UK had a higher level of knowledge about STIs [23]. This might be explained by the effect of cultural differences and religious sensitivities between countries, which limited considerably sexual education and awareness among students. Among the students, 81.6% were aware of cervical cancer. This awareness was lower than the 94.2% obtained in the study conducted by Lenselink et al. (2008) in the Netherlands, however, higher than the 56.4% reported among students in Nigeria and 33.3 % in South Africa (33%) [24–26]. The existence of effective educational programmes to raise awareness of cervical cancer in the European countries might explain the high level of awareness compared to the African countries.

In this study, only 10.45% of the students reported having heard about HPV and showed a poor level of HPV knowledge. This result was lower than a study conducted among Moroccan students (33.7%), but they also showed a poor level of HPV knowledge [21]. This disparity in HPV awareness in the same country can be explained by the fact that students in southern Morocco live in areas with fewer healthcare facilities. Such low HPV awareness has also been reported by several studies, among university students in Nigeria (17.7%) [25], Portugal (55.4%) [27], Malaysia (21.7%) [28], in The Netherlands (17.7%) [24], (50.6%) among female medical students in Ethiopia [29], and they all showed a low level of HPV knowledge. Contrary to this finding, a study carried out in the USA reported that (78.0%) had heard of HPV and showed a relatively high HPV knowledge [30]. Besides that, our finding associated more HPV awareness with being a biology student and having high educational attainment. The study conducted in Morocco by Zouheir et *al*. (2015) agreed that education level had a positive effect on HPV awareness [21]. A similar finding was reported by Rashid et al. (2016) where biology students have shown more awareness towards HPV when compared to non-biology students [31]. A significant association was found between being a health science student and having heard about HPV in the study conducted in Portugal [27]. This had been also agreed by Lenselink et *al*. (2008) where medical students were more aware of HPV than non-medical students [24]. These results suggest a misunderstanding or lack of sexual health background among non-biology students which explains why they showed low HPV awareness compared to biology students. It also seems plausible that students with high educational levels have more chances to hear about HPV than those with lower educational levels. Our finding also revealed that women showed good knowledge about HPV infection than men did, which is consistent with the finding reported by Medeiros et *al*. [27]. The poor HPV knowledge among men highlights that they are less knowledgeable about sexual health issues than women. Indeed, it is very important to intensify HPV knowledge among all the students, especially men because they participate in the transmission and the acquisition of HPV infection.

This present study highlights a poor awareness and knowledge towards STIs in particular HPV infections among university students. Those students who were supposed to be the most knowledgeable showed a big gap in the understanding of their sexual health. Our findings suggest the urgent need for well-designed educational interventions and university campaigns to improve HPV, STIs, and cervical cancer awareness. Targeting higher education students as future educators, parents, and community members will guarantee the next generations with more awareness towards HPV and its negative outcomes.

# Conclusion

In the current study, we showed that attending higher education in Morocco was not sufficient to confer a good knowledge and awareness towards STIs in particular HPV infection. Indeed, the majority of the students 391 out of 479 have heard of cervical cancer, but only 23 out of 479 students were aware that HPV infection is associated with the development of cervical cancer. We found that the most significant factors associated with better awareness about HPV were respectively: (i) the high level of education; (ii) the attendance to curricula related to biological science, while the only associated factor with high knowledge on HPV was the female gender.

Accordingly, we believe that the early incorporation of sexually communicable diseases teaching programs in higher education in Morocco will improve the awareness of young educated people, and reduce the burden of HPV associated diseases among this population at risk.

# References

1.	Lefkowitz EJ, Dempsey DM, Hendrickson RC, Orton RJ, Siddell SG, Smith DB. Virus taxonomy: The database of the International Committee on Taxonomy of Viruses (ICTV). Nucleic Acids Res. 2018;46(D1): D708–17.

2.	Bernard HU. The clinical importance of the nomenclature, evolution and taxonomy of human papillomaviruses. J Clin Virol. 2005;32(SUPPL.):1–6.

3.	Klug SJ, Hukelmann M, Blettner M. Knowledge about infection with human papillomavirus: A systematic review. Prev Med (Baltim). 2008;46(2):87–98.

4.	Burd EM. Human papillomavirus and cervical cancer. Vol. 16, Clinical Microbiology Reviews. 2003. p. 1–17.

5.	Trottier H, Franco EL. The epidemiology of genital human papillomavirus infection. Vaccine. 2006;24(SUPPL. 1): S1/4–S1/15.

6.	Muñoz N, Bosch FX, De Sanjosé S, Herrero R, Castellsagué X, Shah K V., et al. Epidemiologic classification of human papillomavirus types associated with cervical cancer. N Engl J Med. 2003;348(6):518–27.

7.	Smith JS, Lindsay L, Hoots B, Keys J, Franceschi S, Winer R, et al. Human papillomavirus type distribution in invasive cervical cancer and high-grade cervical lesions: A meta-analysis update. Int J Cancer. 2007;121(3):621–32.

8.	Schiffman M, Castle PE, Jeronimo J, Rodriguez AC, Wacholder S. Human papillomavirus and cervical cancer. Vol. 370, Lancet. Elsevier; 2007. p. 890–907.

9.	Bogani G, Leone Roberti Maggiore U, Signorelli M, Martinelli F, Ditto A, Sabatucci I, et al. The role of human papillomavirus vaccines in cervical cancer: Prevention and treatment [Internet]. Vol. 122, Critical Reviews in Oncology/Hematology. Elsevier; 2018. p. 92–7. Available from: https://doi.org/10.1016/j.critrevonc.2017.12.017

10.	Đukić A, Lulić L, Thomas M, Skelin J, Bennett Saidu NE, Grce M, et al. HPV Oncoproteins and the Ubiquitin Proteasome System: A Signature of Malignancy? Pathogens [Internet]. 2020 Feb 18 [cited 2021 Mar 23];9(2):133. Available from: https://www.mdpi.com/2076-0817/9/2/133

11.	Cutts F, Franceschi S, Goldie S, Castellsague X, Sanjose S, Garnett G, et al. Human papillomavirus and HPV vaccines: a review. Bull World Health Organ. 2007;85(9):719–26.

12.	Schiffman M, Wentzensen N. Human papillomavirus (HPV) infection and the multi-stage carcinogenesis of cervical cancer. Cancer Epidemiol Biomarkers Prev [Internet]. 2013;22(4):553–60. Available from: https://www.ncbi.nlm.nih.gov/pmc/articles/PMC3711590/pdf/nihms-449612.pdf

13.	Sung H, Ferlay J, Siegel RL, Laversanne M, Soerjomataram I, Jemal A, et al. Global cancer statistics 2020: GLOBOCAN estimates of incidence and mortality worldwide for 36 cancers in 185 countries. CA Cancer J Clin. 2021;0(0):1–41.

14.	Messoudi W, Elmahi T, Nejjari C, Tachfouti N, Zidouh A, Saadani G, et al. Cervical cancer prevention in Morocco: a model-based cost-effectiveness analysis. J Med Econ [Internet]. 2019;22(11):1153–9. Available from: https://doi.org/10.1080/13696998.2019.1624556

15.	Selmouni F, Sauvaget C, Belakhel L, Lucas E, Khouchoua M, Sankaranarayanan R. Organization and evaluation of a pilot cervical cancer screening program in Morocco. Int J Gynecol Obstet [Internet]. 2016;132(1):25–8. Available from: http://dx.doi.org/10.1016/j.ijgo.2015.06.044

16.	Schiffman M, Castle PE. The Promise of Global Cervical-Cancer Prevention. N Engl J Med. 2005;353(20):2101–4.

17.	Marlow LAV, Waller J, Wardle J. Public awareness that HPV is a risk factor for cervical cancer. Br J Cancer. 2007;97(5):691–4.

18.	Strohl AE, Mendoza G, Ghant MS, Cameron KA, Simon MA, Schink JC, et al. cervical cancer , and HPV vaccinations among African American women. Am J Obstet Gynecol [Internet]. 2015;212:1–5. Available from: http://dx.doi.org/10.1016/j.ajog.2014.06.059

19.	El Sanharawi M, Naudet F. Comprendre la régression logistique. Vol. 36, Journal Francais d’Ophtalmologie. Elsevier Masson; 2013. p. 710–5.

20.	Mouallif M, Bowyer HL, Festali S, Albert A, Filali-Zegzouti Y, Guenin S, et al. Cervical cancer and HPV: Awareness and vaccine acceptability among parents in Morocco. Vaccine [Internet]. 2014;32(3):409–16. Available from: http://dx.doi.org/10.1016/j.vaccine.2013.10.069

21.	Zouheir Y, Daouam S, Hamdi S, Alaoui A, Fechtali T. Knowledge of Human Papillomavirus and Acceptability to Vaccinate in Adolescents and Young Adults of the Moroccan Population. J Pediatr Adolesc Gynecol [Internet]. 2016;29(3):292–8. Available from: http://dx.doi.org/10.1016/j.jpag.2015.11.002

22.	El Gelany S, Moussa O. Reproductive health awareness among educated young women in Egypt. Int J Gynecol Obstet [Internet]. 2013;120(1):23–6. Available from: http://dx.doi.org/10.1016/j.ijgo.2012.07.027

23.	Jones NR, Haynes R. The association between young people’s knowledge of sexually transmitted diseases and their behaviour: A mixed methods study. Heal Risk Soc. 2006;8(3):293–303.

24.	Lenselink CH, Schmeink CE. Young adults and acceptance of the human papillomavirus vaccine. Public Health [Internet]. 2008;122(12):1295–301. Available from: http://dx.doi.org/10.1016/j.puhe.2008.02.010

25.	Makwe CC, Anorlu RI, Odeyemi KA. Human papillomavirus (HPV) infection and vaccines: Knowledge, attitude and perception among female students at the University of Lagos, Lagos, Nigeria. J Epidemiol Glob Health [Internet]. 2012;2(4):199–206. Available from: http://dx.doi.org/10.1016/j.jegh.2012.11.001

26.	Hoque ME. Cervical cancer awareness and preventive behaviour among female university students in South Africa. Asian Pacific J Cancer Prev. 2010;11(1):127–30.

27.	Medeiros R, Ramada D. Knowledge differences between male and female university students about human papillomavirus ( HPV ) and cervical cancer : Implications for health strategies and vaccination. Vaccine [Internet]. 2011;29(2):153–60. Available from: http://dx.doi.org/10.1016/j.vaccine.2010.10.068

28.	Wong LP, Sam IC. Ethnically diverse female university students’ knowledge and attitudes toward human papillomavirus (HPV), HPV vaccination and cervical cancer. Eur J Obstet Gynecol Reprod Biol. 2010;148(1):90–5.

29.	Tesfaye ZT, Bhagavathula AS, Gebreyohannes EA, Tegegn HG. Knowledge and awareness of cervical cancer and human papillomavirus among female students in an Ethiopian University: A cross-sectional study. J Basic Clin Pharm [Internet]. 2018 [cited 2021 Mar 24];9(1):22–5. Available from: www.jbclinpharm.org

30.	Gerend MA, Magloire ZF. Awareness, Knowledge, and Beliefs about Human Papillomavirus in a Racially Diverse Sample of Young Adults. J Adolesc Heal. 2008;42(3):237–42.

31.	Rashid S, Labani S, Das BC. Knowledge, awareness and attitude on HPV, HPV vaccine and cervical cancer among college students in India. PLoS One. 2016;11(11):1–11.
