# Supplementary material for: Awareness and knowledge associated to Human papillomavirus infection among university students in Morocco: A cross-sectional study
Source: PLoS One. 2022 Jul 8;17(7):e0271222. doi: 10.1371/journal.pone.0271222 (PMC9269923; doi:10.1371/journal.pone.0271222)
Supplement: Attachment — Submitted filename: Response to Reviewers.docx [file pone.0271222.s004.docx]

Dear Muhammad Tarek Abdel Ghafar,

I would like to thank you and the reviewers for the useful comments on our article. I now have edited the manuscript to address the concerns raised by the reviewers. Accordingly, please find below the response, point by point.

I hope that the manuscript, in its actual form, meets the standards for publication in PLOS ONE.

I am looking forward to hearing from you soon,

Sincerely yours,

EL MANSOURI Nezha.

**Academic editor:**

**Point 1:**

**Please ensure that your manuscript meets PLOS ONE's style requirements, including those for file naming.**

Corrected. We hopefully have no divergences from the style requirements now.

**Point 2:**

**Please provide additional details regarding participant consent. In the ethics statement in the Methods and online submission information, please ensure that you have specified what type you obtained (for instance, written or verbal, and if verbal, how it was documented and witnessed).**

We amended the ethics statements to emphasize that all participants gave written consent, line 136-137 (Simple Markup track changes).

An informed written consent was obtained from each participant when they agreed to be included in this study. The ethics statement was also amended in the submission form.

**Point 3:**

**We note that you have stated that you will provide repository information for your data at acceptance. Should your manuscript be accepted for publication, we will hold it until you provide the relevant accession numbers or DOIs necessary to access your data. If you wish to make changes to your Data Availability statement, please describe these changes in your cover letter and we will update your Data Availability statement to reflect the information you provide.**

Thank you for the remark.

I would like to let you know that the box referring to “the additional data availability information” was ticked by mistake during the submission online. We removed this tick mark. Instead, all relevant data are now available in the manuscript and in the supporting information files.

**Reviewer #1**

**The manuscript is well written and has potential. However, a few typographical and technical errors have been noted in the attached document.**

All highlighted typographical and technical errors have been corrected. We thank the reviewer for helping us.

**Some highlights to address:**

**Point 1:**

**Please clearly state your sample size**

Amended, we added the following sentence in line 92 (Simple markup track changes): A total of n= 479 participants were selected using a simple random selection process.

**Point 2:**

**Maintain one style of citation**

Amended.

**Reviewer #2**

**Point 1:**

**The manuscript is well presented and written in standard comprehensible English. However, it can still benefit from structural rearrangement of some of the sentences to make readability easier, especially the result and discussion section.**

Amended.

**Point 2:**

**It was mentioned that the study data is available without restriction but information on where and how it could be accessed was not provided.**

We have sent the study data (Excel file) as a supporting information file.

**The section on material and methods:**

**Point 3:**

**More clarity should be provided about the mode of questionnaire administration. How was this done? (self-administration, facilitated or online assessment). This need to be clear. The section on the questionnaire could probably focused more on the description of the questionnaire and approach to the response which are more useful for replicability rather than on specific content of the questionnaire.**

The administration of the questionnaire was a face-to-face interview questionnaire done by our team members, who also helped in clarifying questions for the students. We added a sentence in the manuscript that emphasize the type of questionnaire and how it was implemented on line 99 (simple markup track changes).

**Reviewer #3**

**Point 1:**

**Title: Knowledge and Awareness toward Human Papillomavirus and Cervical Cancer (CC): A Cross-sectional Study Among University Students in Morocco. This does not suggest that you will find the factors associated with awareness and knowledge about HPV and CC.**

Thank you for your comment. We have changed the title to reflect more the study objectives.

Corrected title: “Human Papillomavirus knowledge, awareness and associated factors: A cross-sectional study among university students in Morocco.”

**Point 2:**

**Introduction: To what extent are university students at risk for HPV related infections such as cervical cancer? This information can justify your research if included in the introduction.**

Cervical cancer is a consequence of the persistence of HPV infection which is the most common sexually transmitted infection worldwide. The highest rates of HPV infection are found among young and sexually active men and women. It was also shown that HPV infection prevalence decreased with increasing age from a peak prevalence in younger women (≤25 years of age) (Smith JS et al., Age-Specific Prevalence of Infection with Human Papillomavirus in Females: A Global Review. J Adolesc Heal. 2008 43:5–25). Students at the university are young adults and at the age of sexual activity, and the probability of contracting HPV infection during this period is high. We have added this information in the introduction on lines 52-53 and 87 (simple markup track changes).

**Point 3:**

**Methods section: how do calculate the sample size of 479 students and allocate in to faculities and lebow? Describe the sample size determination and prcudure.**

We have added information on the manuscript to clarify the sample size calculation, lines 93-97 (simple markup track changes). As follow:

The size of the sample was calculated based on a 5.0% error range, a 95% confidence interval (CI) for a total population of 7000 students in the faculty of sciences, university Ibn Zohr. With an anticipated proportion of knowledge and awareness on HPV deficiency of 50%. The sample size was calculated using the website OpenEpi <https://www.openepi.com/Menu/OE_Menu.htm>. The minimal sample size required for the study was 365 persons.

**The way you describe measurement of knowledge in your research is interesting. However, the cut of point 3 correct answers needs a reference or justification?**

We have added information in the manuscript to clarify the cut of point 3, on lines 115-121 (simple markup track changes), as follow:

We allocated one point for each correct answer, and zero for the incorrect or the “Don’t know” responses. The total score was calculated by adding up all scores for the five questions (Q4 to Q8), and possible scores range from 0 to 5. The scores higher than the median (median=2) (Higher or equal than 3 correct answers) were designated as categories of “High knowledge”. By contrast, “Low knowledge” referred to scores equal or lower than the median or (Lower than 3 correct answers) (Tusimin M et al. Sociodemographic determinants of knowledge and attitude in the primary prevention of cervical cancer among University Tunku Abdul Rahman (UTAR) students in Malaysia: Preliminary study of HPV vaccination. BMC Public Health. 2019;19(1):1–6).

**Point 4:**

**Results: The presentation of your result shall be according to your objectives. For example:**

**a) describe sociodemographic and reproductive characteristics of your participants**

**b) their awareness about HPV and CC**

**C) Their knowledge about HPV and CC**

**D) Factors affecting their knowledge**

**So table 1 need to be split**

We thank the reviewer for the constructive feedback. All the suggested changes in the results section structure and table 1 have been made. Table 1 is split into Tables 1 and 2, on pages 7 and 8 respectively.

However, regarding point (c), our work was mainly focused on what causes CC, instead of the knowledge toward CC.

**Why class year is not recorded? as long as awareness of the first year and graduating class student won't be the same**

We did record class year for each student during the data collection as follow: 1^st^ year, 2^nd^ year, 3^rd^ year and masters, and then we considered (1^st^ year, 2^nd^ year, 3^rd^year = undergraduate and masters= graduate). Indeed, logistic regression is the type of analysis to use when we work with binary data. Therefore, we categorized all the independent variables in our study into two groups. We omitted this details in the manuscript in order to keep the independent variable (Level of education) dichotomous.

**On table2: the OR for age and level of study are misplaced. correct them. again the instead of writing ref, use 1**

Corrected.

**In table2: the uni and multivariate analysis result should be presented separately if you must report. For me Univariate is not needed. The p- value and confidence interval should be reported in the multivariate analysis. You need to present also ont only about HPV but also CC. In general table 2 cannot be interpreted correctly, there are missing elements. i.e the CI should be consistent with the response of the outcome variable. This needs revision.**

As required above, we have modified the table accordingly (Table 2 is now Table 3 on page 10).

**References: Replace references older than 2017 for article or 2002 for books with recent ones**

As required, the references were updated in the introduction section.

**Reviewer #4**

**Major comments**

**Point 1:**

**“The participants were selected using a simple random selection process. However, the population of students targeted was mainly those attending courses in the Faculty of Sciences.” (page 4, lines 20-21). The two sentences contradict each other. The source population is unclear; therefore, the study population seems to be not representative for any population group.**

Thank you for your comment. We have corrected this in the manuscript on line 90-92 as follow:

- This cross-sectional study was conducted on the site of the faculty of sciences, Ibn Zohr University (IZU), in Agadir, Morocco, where the majority of the students were attending their curricula. A total of n= 479 participants were included using a simple random selection process.

- The choice of the population: In fact, university students are young adults and at the age of sexual activity, making them at high risk of contracting HPV infection.

**Point 2:**

**“This was followed by an open-ended question with “Yes” or “No” response options…” (page 5, lines 6-7). A question with Yes/No options is not an open-ended question.**

Corrected in the manuscript on lines 106, 108 and 112 (Simple markup track changes).

**Point 3:**

**The development of the survey tool is not explained. Is it a standardized questionnaire? Was it pilot tested? Were questions from other surveys adopted?**

The questionnaire is a combination of items adapted from previously published studies below:

- Zouheir Y et al., Knowledge of Human Papillomavirus and Acceptability to Vaccinate in Adolescents and Young Adults of the Moroccan Population.

J Pediatr Adolesc Gynecol. 2016;29(3):292–8;

- Rashid S et al., Knowledge, awareness and attitude on HPV, HPV vaccine and cervical cancer among the college students in India.

PLoS One. 2016;11(11):1–11.)

We added this modifications in the manuscript on lines: 99-101.

**How was the cut-off level of knowledge determined?**

We have added information in the manuscript to clarify the cut of point 3, on lines 115-121, (simple markup track changes), as follow:

We allocated one point for each correct answer, and zero for the incorrect or the “Don’t know” responses. The total score was calculated by adding up all scores for the five questions (Q4 to Q8). The possible scores range is from 0 to 5. The scores higher than the median (median=2; Higher or equal than 3 correct answers) were considered as categories of “High knowledge”. By contrast, “Low knowledge” referred to scores equal or lower than the median or (Lower than 3 correct answers) (Tusimin M et al. Sociodemographic determinants of knowledge and attitude in the primary prevention of cervical cancer among University Tunku Abdul Rahman (UTAR) students in Malaysia: Preliminary study of HPV vaccination. BMC Public Health. 2019;19(1):1–6)).

**Point 4:**

**Why was age dichotomized and not handled as a continuous variable? This approach introduced loss of information.**

Corrected. The independent t-test was performed when we added age as a continuous variable (Table 1 and Table 2.). However, in the logistic regression analysis age was dichotomised based on the mean to facilitate the analysis (Table 3).

**Point 5:**

**How could it happen that fewer participants knew the association between HPV and cervical cancer than those who knew that HPV is its leading cause? It seems a contradiction.**

The present study reveals that 60.4% of the students who have heard about HPV infection, did show a low knowledge about the virus itself. Those students, through their responses, showed that there is a gap in their understanding of this infection and of its consequences (i.e. association between HPV and CC). In addition, due to the nature of the answers proposed (i.e. Yes/ No/ Don’t know) we might have overestimated the level of knowledge of our surveyed population. This may constitute one of the limitations of this study. Indeed, open questions are needed to avoid influencing the answers given by the participants.

**Point 6:**

**Age essentially correlates with the level of education; therefore, it is questionable whether age can be regarded as an independent risk factor functioning as confounder in the awareness-level of education relationship.**

We agree that level of education correlate with age; this has been emphasized in the discussion section, on lines 247-250 (Simple markup track changes).

**Minor comments**

We thank the reviewer for spotting these errors and for their constructive feedback. All the suggested minor comments have been corrected and highlighted in the manuscript (red font).
