# Supplementary material for: Awareness and knowledge associated to Human papillomavirus infection among university students in Morocco: A cross-sectional study
Source: PLoS One. 2022 Jul 8;17(7):e0271222. doi: 10.1371/journal.pone.0271222 (PMC9269923; doi:10.1371/journal.pone.0271222)
Supplement: Attachment — Submitted filename: Response to Reviewers.docx [file pone.0271222.s005.docx]

Dear Muhammad Tarek Abdel Ghafar,

I would like to thank you and the reviewers for the useful comments on our manuscript. I now have edited the manuscript to address the concerns raised by the reviewers. Accordingly, please find below the response, point by point.

I hope that the manuscript in its actual form meets the standards for publication in PLOS ONE.

I am looking forward to hearing from you soon,

Sincerely yours,

EL MANSOURI Nezha.

**Academic editor:**

**Please review your reference list to ensure that it is complete and correct.**

Amended. Thank you.

we have completed the missing information for each reference. We replaced the reference number [13] on lines 307-309 ( (simple markup track changes) : OpenEpi:Sample Size for X-Sectional,Cohort,and Clinical Trials [Internet]. [cited 2022 Feb 21]. Available from: <http://www.openepi.com/SampleSize/SSCohort.htm>, with Sullivan KM, Dean A, Minn MS. OpenEpi: A web-based epidemiologic and statistical calculator for public health. Public Health Reports. 2009; 124(3): 471–474. doi: [10.1177/003335490912400320](https://dx.doi.org/10.1177%2F003335490912400320)

**Reviewer #2**

**Need for a minor correction at Line 105. A reference to a "Yes" or "No" question is not open ended.**

Amended. Thank you.

**Reviewer #3**

**Point 1**

**Knowledge comes after awareness.**

Amended

Corrected title: “Awareness and knowledge associated to Human Papillomavirus infection among university students in Morocco: A cross-sectional study”.

**Point 3**

**Analysing the associated factors for awareness and knowledge is just repetition.**

In the present study awareness and knowledge related to HPV infection were investigated separately, as well as the associated factors. Awareness on HPV was investigated using a Yes or No question “Have you ever heard of Human Papillomavirus (HPV)? This question was asked to the whole population included in this study. By contrast, the knowledge related to HPV infection was investigated exclusively among students that responded “Yes” to the previous question (i.e. Have you ever heard of Human Papillomavirus?)

**Point 3**

**The tabular presentation needs correction.**

**The logical order of presentation should be according to your objectives.**

**First description of study participants, then knowledge, then the factors. The others are not relevant. Try to describe what is the level of knowledge about HPV/CC and the associated factors.**

We investigated the level of knowledge related to HPV infection and the factors that might be associated. However, the adopted questionnaire did not investigate specifically the level of knowledge related to cervical cancer. Indeed, our investigation focused on awareness and knowledge related only to HPV infection and the factors that might be associated.

**The Chi Square is not important. you already categorise your dependent variable knowledge as high and low. So, you can use logistic regression.**

Amended on lines 212-214 (simple markup track changes), and in table 2.

Thank you.
