# Supplementary material for: Awareness and knowledge associated to Human papillomavirus infection among university students in Morocco: A cross-sectional study
Source: PLoS One. 2022 Jul 8;17(7):e0271222. doi: 10.1371/journal.pone.0271222 (PMC9269923; doi:10.1371/journal.pone.0271222)
Supplement: Attachment — Submitted filename: Response to Reviewers.docx [file pone.0271222.s006.docx]

Dear Muhammad Tarek Abdel Ghafar,

I would like to thank you and the reviewers for the useful comments on our manuscript. I now have edited the manuscript to address the concerns raised by the reviewers. Accordingly, please find below the response, point by point.

I hope that the manuscript in its actual form meets the standards for publication in PLOS ONE.

I am looking forward to hearing from you soon,

Sincerely yours,

EL MANSOURI Nezha.

**Academic editor:**

**Please cite the supplementary tables within the text.**

Amended, we cited the supplementary tables on lines 142-143, 187 and 200 (simple markup track changes).
